# User-Centered Design and Usability of a Culturally Adapted Virtual Survivorship Care App for Chinese Canadian Prostate Cancer Survivors: Qualitative Descriptive Study

**DOI:** 10.2196/49353

**Published:** 2024-01-01

**Authors:** Karen Young, Ting Xiong, Rachel Lee, Ananya Tina Banerjee, Myles Leslie, Wellam Yu Ko, Quynh Pham

**Affiliations:** 1 Centre for Digital Therapeutics Techna Institute University Health Network Toronto, ON Canada; 2 Institute of Health Policy, Management and Evaluation Dalla Lana School of Public Health University of Toronto Toronto, ON Canada; 3 Department of Epidemiology, Biostatistics and Occupational Health McGill University Montreal, QC Canada; 4 School of Public Policy University of Calgary Calgary, AB Canada; 5 Men’s Health Research Program University of British Columbia Vancouver, BC Canada; 6 Toronto General Hospital Research Institute University Health Network Toronto, ON Canada; 7 Telfer School of Management University of Ottawa Ottawa, ON Canada; 8 School of Public Health Sciences University of Waterloo Waterloo, ON Canada

**Keywords:** digital health, virtual care, digital therapeutics, prostate cancer, cancer survivorship, user-centred design, usability, supportive care, cultural adaptation, Chinese Canadians

## Abstract

**Background:**

Cultural adaptations of digital health innovations are a growing field. However, digital health innovations can increase health inequities. While completing exploratory work for the cultural adaptation of the *Ned* Clinic virtual survivorship app, we identified structural considerations that provided a space to design digitally connected and collective care.

**Objective:**

This study used a community-based participatory research and user-centered design process to develop a cultural adaptation of the *Ned* Clinic app while designing to intervene in structural inequities.

**Methods:**

The design process included primary data collection and qualitative analysis to explore and distill design principles, an iterative design phase with a multidisciplinary team, and a final evaluation phase with participants throughout the design process as a form of member checking and validation.

**Results:**

Participants indicated that they found the final adapted prototype to be acceptable, appropriate, and feasible for their use. The changes made to adapt the prototype were not specifically culturally Chinese. Instead, we identified ways to strengthen connections between the survivor and their providers; improve accessibility to resources; and honor participants’ desires for relationality, accountability, and care.

**Conclusions:**

We grounded the use of user-centered design to develop a prototype design that supports the acts of caring through digital technology by identifying and designing to resist structures that create health inequities in the lives of this community of survivors. By designing for collective justice, we can provide accessible, feasible, and relational care with digital health through the application of Indigenous and Black feminist ways of being and knowing.

## Introduction

Digital health has been posited as a pathway to more equitable and holistic care [1,2]. However, the digital divide, or the capacity for digital technology to exacerbate inequities, has been widely described [3]. Its differential impacts on the social determinants of health are known as the digital determinants of health [4]. Recent years have seen an acceleration of digital health innovations (DHIs) such as digital therapeutics into health care systems, which was supercharged by the COVID-19 pandemic and the resulting widespread implementation of telemedicine [4]. One such digital therapeutic is the *Ned* Clinic (“No Evidence of Disease”), which aims to optimize clinical care and patient self-management through virtual asynchronous care delivery for prostate cancer (PCa) survivors [5]. The *Ned* Clinic platforms, including clinician-led (*Specialist Ned*) and nurse-led (*Ned Nurse*) interventions, were developed at the University Health Network in Toronto, Canada, by a consortium of stakeholders [5].

PCa is the most commonly diagnosed nonskin cancer for Canadian male individuals, and most (99%) are estimated to be diagnosed in male individuals aged 50 years and older [6]. Older adults are negatively impacted by the digital divide [7]. Race, a social determinant of health, is also linked to worse survivorship and care outcomes for PCa survivors, most notably for Black male individuals [8]. Asian (generally defined as East Asian and South Asian ethnicity) male individuals have been found to have better survival rates than the median but are more likely to present with advanced PCa, suggesting systemic issues with identifying health issues and obtaining timely appropriate care [9]. These differences carry over into the delivery of follow-up care, as PCa survivors’ care needs and access to care are affected by the complex intersection of ethnicity, culture, and other social and structural factors [10,11].

Cultural adaptation is the process of applying changes to existing health interventions based on “surface” (social and behavioral characteristics) and “deep” (worldview, norms, beliefs, and values) cultural structures [12]. As these structures are known to impact beliefs about illness and well-being, the intent is to provide intervention benefits for communities that have experienced health inequities [13]. Culturally adapted DHIs appear to have been most widely reported in the field of mental health; in contrast, cultural adaptations of cancer survivorship apps have not been published, likely owing to the few interventions in this area [2,14]. The frameworks that appear to be most widely used to adapt health interventions were developed by Bernal et al [15], Resnicow et al [16] (an adaptation of the model by Bernal et al [15]), and Barrera and Castro [17].

However, these guidelines and models often use framings of cultural sensitivity and competency (eg, Resnicow et al [16] and Castro et al [18]), continuing to place the burden of change on individuals rather than addressing the upstream structural determinants of health. These framings can serve to “museumize” and problematize identity categories and culture as causes of ill-health, echoing the long-standing use of culture as a scapegoat to fault specific communities for health inequities. Moreover, defining “culture” for such adaptations can be a complex process in Canada, where culture, race, ethnicity, settler colonialism, and white supremacy (ie, the social and structural determinants of health) all create intersectional and differential lived experiences under a putatively shared identity—Canadian [19-21].

This research reports on the second and final phase of a project to design a cultural adaptation of the patient-facing *Ned* Clinic virtual follow-up care app for Chinese Canadian PCa survivors. In phase 1, we completed formative work distilling a set of themes relevant to survivors’ user needs for follow-up and virtual care. Following the user-centered design (UCD) framework, we describe the results of the design and formative evaluation of a culturally adapted prototype of the app.

## Methods

### Study Design

The overall qualitative descriptive study design was structured using the community-based participatory research (CBPR) and UCD frameworks [22-24]. This study was conducted at the University of Toronto between December 2022 and March 2023 during the COVID-19 pandemic. For communities that face barriers to care, it was found that CBPR practices such as our engagement of a key informant and invitations to community members to share their lived experiences through open-ended interviews are appropriate [1,25]. CBPR concepts were applied to meaningfully involve the community (including several authors of this study) and return the results for their benefit. Here, *community* represents a “symbolic totality as well as a practical multiplicity,” as the Chinese Canadian community is highly heterogeneous [26]. We view our participants as a coalition of self-identified Chinese Canadian individuals impacted by PCa survivorship to attend to their differences.

The Chinese Canadian community is an immigrant community that exists as a result of settler colonialism. In recognizing this, we redefine “immigrants” as “people with ancestral roots outside of Indigenous lands, who are beholden to Indigenous laws and epistemologies” [27]. This definition led us to apply a relational paradigm to this project and an axiology of relational accountability. It also provided a pathway to apply several multilevel Indigenous and Black feminist theorizations, guiding principles, and tools [27-29]. These included decolonial theory, *Etuaptmumk* (two-eyed seeing), intersectionality, and cultural safety to inform our conceptualization of digital space as intimately related to land [27,30,31]. This approach allowed us to contextualize the place-related experiences of our participants and uncover their desires for relational and culturally safe care [32]. We noted that these desires are not specifically Chinese, and this presented an opportunity to design for relationally connected digital health.

UCD is a flexible, iterative, and evidence-based 3-step design process framework that consults, involves, and considers the needs of the end user throughout the entire project [23]. Phase 1 of this study encompasses steps 1 and 2; phase 2 encompasses steps 2 and 3. We present this study according to the Consolidated Criteria for Reporting Qualitative Research (COREQ) guidelines [33].

### Step 1: Ideation and Concept Generation

To contextualize the potential use of this app, we sought to understand the structures that impact Chinese Canadian PCa survivors’ experiences with follow-up care and virtual care. The results of this phenomenologically informed exploratory-descriptive qualitative study are described elsewhere [34]. Based on the findings of this formative research, we synthesized a list of design principles (Table 1), which we then categorized into the cultural adaptation taxonomy created by Spanhel et al [14] to systematically adapt the patient-facing prototype.

**Table 1:** Summary of design principles for the adaptation of the *Ned Nurse* patient-facing app.

Research finding	Design principle	Taxonomic classification [14]^a^
PHI^b^ freedom: patients felt that they were expected to track and remember overwhelming amounts of information.	The system should automatically update, store, and provide access to PHI on demand.	Content components: (9) Goals of treatment (10) Methods of treatment
Access to personalized education and information: patients felt that they were unable to access information about their care options and disease status.	The system should provide access to personalized and evidence-based information regarding staging, self-management, and treatment options.	Content components: (9) Goals of treatment (10) Methods of treatment
Continuity of care: patients desired a connection with their provider and the ability to communicate during times of need.	The system should improve accessibility and continuity of care, as strong care relationships create a sense of safety.	Content components: (9) Goals of treatment (10) Methods of treatment
Security: patients expressed suspicion about digital health because they had concerns about surveillance and security.	The system should be architected and built with a high level of security and privacy.	Methodological components: (12) Functionality
Accessibility: patients wanted to access care in readable and accessible language formats.	The system should provide readable language and accessible language formats.	Content components: (5) Language translation (6) Language tailoring
Digital literacy: patients felt comfortable with their device of choice but desired simplicity, form over function, and accessible help and documentation.	The system should prioritize usability, provide straightforward instruction and support, and maintain simple user interface and user experience design.	Methodological components: (11) Structure (12) Functionality (13) Design and aesthetics
Care coordination: patients felt like they were expected to coordinate their care, as communication between specialists, primary care, and other services were fragmented.	The system should coordinate and provide a clear follow-up appointment schedule.	Content components: (9) Goals of treatment (10) Methods of treatment
Resources: patients felt unable to access, refused, or unaware of needed resources such as mental health support.	The system should provide accessible pathways to resources, such as psychological support, supportive care, and financial support.	Content components: (8) Difference in concepts of mental health and its treatment (9) Goals of treatment (10) Methods of treatment

^a^The design principles used to adapt the *Ned* Clinic patient app identified here are classified to the corresponding taxonomic components found in Spanhel et al [14].

^b^PHI: personal health information.

### Step 2: Design and Development

**Figure 1:**
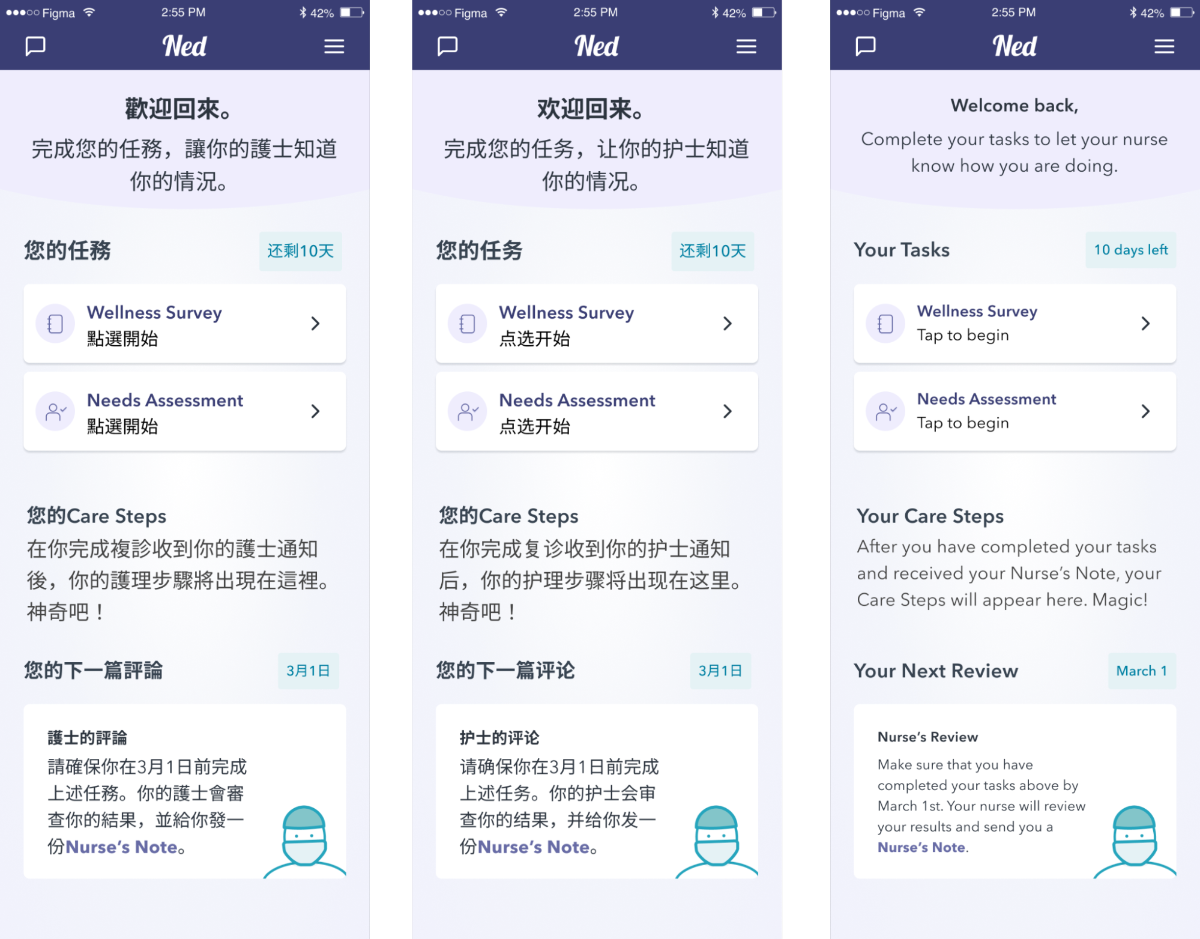
Wireframes of the adapted *Ned Nurse* homepage in all 3 language versions.

We applied these design principles to adapt the *Ned Nurse* patient app for Chinese Canadian survivors. A composite profile of a sample representative user was created to situate the design team during the development of the wireframes. A list of 5 use scenarios was created to guide the adaptation. These scenarios encompassed the design principles created in step 1 and included actions such as completing follow-up tasks, accessing a follow-up care schedule, and using the app to chat with a clinician. All use scenarios are described in the interview guide (Multimedia Appendix 1). Then, the original *Ned Nurse* app wireframes were redesigned to reflect the features required to operationalize these scenarios through the app, resulting in a new prototype. The prototype was created in Figma (Figma Inc.) on an iPhone 13 (Apple Inc) interface. This initial adaptation was iteratively critiqued by a team of researchers and human factors designers to refine the content, user interface, and user experience. Once the adapted prototype was finalized, it was translated from English into written Chinese via the translation process outlined in Haldane et al [35]. This resulted in 3 versions of the adapted prototype in English, Simplified Chinese, and Traditional Chinese. The app home page in each language version is shown in Figure 1.

### Step 3: Evaluation

#### Overview

We empirically evaluated the acceptability, appropriateness, and feasibility of the adaptation through a cultural safety lens [22]. These dimensions are early-stage implementation outcomes and have also been found to be core to the success of DHIs [36,37]. A moderated cognitive walkthrough approach and the think-aloud protocol were used to construct a semistructured interview guide encompassing the 5 scenarios describing usual tasks that an end user might complete through the app [38].

#### Usability Testing

Facilitators began each test by outlining the usability testing procedure and think-aloud protocol. Context regarding the intended use and deployment of the *Ned Nurse* system was provided. Participants were asked to complete a series of actions for each scenario on the prototype to evaluate its design and functionality. We asked participants to think and speak about improvements they desired during their evaluations. In situations where the participant was unable to access the prototype on their device, they were asked to state their intended actions using the think-aloud protocol to the facilitator, who completed the action in the prototype on their behalf.

Interviews were completed through Microsoft Teams or Zoom (Zoom Technologies Inc). Informed consent for this work was previously obtained as part of overall study consent from participants. Participants were provided with the choice of completing their interview in Cantonese, Mandarin, or English and were also able to choose which language they wished to test the prototype in. The results of each usability test were iteratively analyzed via content analysis. Audio recordings of the participant interviews were translated into English as needed, according to the translation process described previously. A deductive and inductive content analysis approach was used, in which analysis of the data was completed by coders (TX and KY) through a process including open coding, creating categories, and abstraction [39]. Recommendations were applied in real time to create a final prototype that incorporated feedback from each user over the course of usability testing.

### Positionality

An important marker of excellent qualitative research is “sincerity” or positionality, which indicates that the researcher has thought about and is reflective and aware about their values, experiences, biases, and inclinations within their research [40]. Here, the lead researcher reports on their social position, personal experiences, and political and professional beliefs to center the active role that the researcher plays in the framing of the research problem, interpretation of data, methods used, and the reporting of the results [41].

KY is a health informatics trainee and second-generation Chinese Canadian settler who was born and raised in the Greater Vancouver Regional District (GVRD) by a working-class, first-generation immigrant family with roots in southeastern China. She does not have any direct experience with PCa and has not previously provided care for PCa survivors. KY works primarily from a relational paradigm, focusing on the structures, contexts, and relationships that shape the design, development, and implementation of digital therapeutics and health technologies. She led and participated in all study activities.

### Setting and Place

This study was conducted in the GVRD, located on the current, unceded, and future territories of the 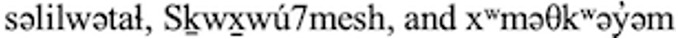 (Tsleil-Waututh, Squamish, and Musqueam) First Nations. The GVRD is home to one of Canada’s oldest and largest living Chinese communities, including persons and families whose stories and identities span multiple geographies and generations [42]. The lead (KY) and senior author (QP) established relations with a supportive care program that provides care for Chinese Canadian PCa survivors and a Chinese PCa support group in this area. A key community informant agreed to guide this study and review and approve study materials.

### Ethical Considerations

Research ethics approval for this study was obtained from the University of Toronto research ethics board (Human Protocol #43145). Written and verbal informed consent to participate in both phases of the project was obtained from all participants prior to interviews via the REDCap tool (Research Electronic Data Capture; Vanderbilt University), hosted at the University of Toronto. All data collected and disseminated here have been de-identified. Participants were provided with an honorarium of $50.00 CAD ($37.65 USD) per hour in appreciation of their time.

## Results

### Demographics

Usability testing was performed by 6 user testers, convenience sampled from the pool of 14 survivors and partner-caregivers who participated in the first phase of work as a form of member checking. This sample was also informed by Nielsen-Norman usability testing guidelines [43]. The reasons for nonparticipation were not collected. To protect the privacy of the participants involved in this phase, a demographic overview of the overall research project is provided here. Of the 14 participants in the first phase of this project, all survivors identified as men (n=12, 86%), and all partner-caregivers identified as women (n=2, 14%). A total of 13 (93%) participants indicated that they spoke English as an additional language. Most made an income between CAD $15,000 (US $11,048) and CAD $100,000 (US $73,653; n=12, 86%), lived in an urban area (n=13, 93%), were married (n=12, 86%), were educated beyond high school (n=13, 93%), and were retired (n=9, 64%). A 50/50 split emerged between preferences for smartphone or desktop or laptop use. Most (n=10, 71%) self-rated as being comfortable with their device. Participants indicated that they had 2 or fewer smartphone health apps (n=13, 93%).

### Phase 1: Ideation

Table 1 summarizes the user requirement findings that emerged from previous formative research in phase 1 of this project and their subsequent translation to design principles.

### Phase 2: Design and Development

#### Overview of Ned Nurse

An overview of the *Ned Nurse* clinical trial protocol is described by Pham et al [5]. The findings from formative work on the perspectives of health care providers, patients from the wider PCa survivor community, and the service design of the platform are forthcoming. Briefly, *Ned Nurse* digitally operationalizes a nurse-led model of survivorship care. Patients complete a series of tasks or access resources designed to support them in their survivorship. The platform aims to facilitate holistic care for patient quality of life.

#### Overview of the Adapted Patient-Facing System

The patient-facing adaptation set 2 user-input “care tasks,” a validated questionnaire (Expanded Prostate Cancer Index Composite-Clinical Practice [EPIC-CP]) and a needs assessment survey, to constitute a single *Ned Nurse* “review” [5,44]. Language within the app avoided wording such as appointment, visit, and so forth to clarify the differences between synchronous and asynchronous care encounters. The user interface and user experience were designed to draw the user’s attention to these tasks on the homepage immediately after login. All features were accessible via an in-app hamburger menu.

User inputs to the questionnaire were triaged via a decision-tree algorithm [45]. The algorithm was designed to return in-app self-management resources within a progress note (“Nurse’s Note”) automatically available to the user after input submission. If the algorithm detected that the patient required further support, they were prompted to specify domains for follow-up and asked to select their preferred contact method. This action would flag this patient to the nurse for follow-up. Resource links would appear on the homepage after the note was read and cleared.

To ensure that patients were aware of their review schedule, a feature was designed to display the last date, frequency, and next date of their expected reviews. The name of the nurse in charge and an explanation of their *Ned Nurse* role were provided to strengthen the perceived connection between the user and the nurse. This feature also set expectations for manual response times and included a link to users’ previous submissions for on-demand access.

Resources were made available in 3 separate categories: symptom self-management advice, PCa information and education, and support and programmatic resources. Within each category, resources were further categorized. For example, symptom management included resources for symptoms such as anxiety, urinary incontinence, and hot flashes. Each resource provided an overview; relevant self-management steps; off-app links; and the ability to email, print, or save the resource. The feature home page also sectioned resources saved by the user (“Saved Resources”) and resources picked for the user (“Picked for Me”) by their nurse.

All available and historical prostate-specific antigen and testosterone blood work results were made available in chronological order to the user on-demand in a separate feature. Finally, a chat feature was designed to explore whether users might find it useful. It incorporated both responses in English from an automated support assistant (chatbot) and manually submitted by the nurse. This feature was simulated for evaluation.

### Phase 3: Evaluation

Of the 6 participants, 2 (33%) tested in Cantonese, 3 (50%) tested in English, and 1 (17%) tested in Mandarin. These ratios correspond to testing of the Traditional Chinese, English, and Simplified Chinese versions. We note that patients who completed their testing in 1 language were functional to fluent in 1 or all of the other languages and provided critique for multiple versions.

Overall, there was strong agreement that the adaptation presented here would be acceptable, appropriate, and feasible for use, with the exception of the chat feature. Participants agreed that this app would make them feel comfortable and safe by allowing them to have more control over their care, access to resources, and stronger connections to their providers. They were encouraged by its perceived ability to meet their needs by protecting their connection with their providers, leveraging the functional flexibility of digital health, and providing resources beyond what they currently accessed. It was particularly valuable that features could be accessed at their convenience, as some felt that their follow-ups were far too short to meet their needs. Overall, 5 (83%) of 6 participants indicated that the level of support provided by this app was beneficial enough that it should be offered to patients prior to beginning treatment, or even at the point of diagnosis.

Participants’ critiques centered on expanding flexibility, access to information, and streamlining responses. They felt that responses for some assessment questions (from 4 to 8 options) were overwhelming and should be reduced (3/6, 50%). English-Chinese translations would increase self-confidence in navigating the health care system. Medication names were spotlighted as particularly difficult. This was noted as an opportunity to expand the app’s personal health information (PHI) storage, as a feature containing self-reported PHI (including medications) would be helpful to reference. Pictures and videos were desired instead of textual explanations. Laboratory results were asked to be displayed graphed or with severity indicators by 1 participant, and a text size adjustment function was requested by another.

Support for sexual dysfunction was not requested explicitly but appeared to be implied (3/6, 50%). A sexual therapy resource section was requested by 1 participant. Another noted that they would be more comfortable with nurses gendered as men as they felt uneasy when discussing sexual dysfunction with women. A final participant was keen to indicate that sexual dysfunction was a major area of concern when completing the EPIC-CP questionnaire.

As resources could be accessed on demand, some indicated that more would be beneficial. However, other participants expressed that the number displayed in the prototype were more than sufficient, reflecting our previous study findings on the bifurcated information-seeking behaviors of Chinese Canadian PCa survivors. Participants were also asked if they might find having their imaging results helpful. Although the majority (4/6, 67%) said no, those who said yes (2/6, 33%) were keen on having this information, especially if they needed to travel outside of Canada.

The questionnaire and assessment were generally deemed to be acceptable by most participants (4/6, 33%), with several notable dissents (2/6, 33%). The EPIC-CP question regarding hormonal function was highlighted as confusing by some because the connection between hormonal function and fatigue was not readily apparent. The spiritual domain in the needs assessment was flagged, as some thought that it would not be appropriately addressed by the nurse. Those who felt uncomfortable with this domain noted that they would prefer speaking about these needs to a spiritual leader. Agreement on appropriate response times also varied.

The chat function was deemed possibly helpful but likely unnecessary (4/6, 67%). As all chat interactions were in English, participants who were not confident in their English communication skills felt that their use of this feature would be limited (3/6, 50%). Others felt reminded of troubleshooting cable services rather than feeling connected to their provider. It was emphasized that any opportunity to improve connections to their providers through the app would be appreciated.

## Discussion

### Principal Findings and Implications

This study provides an applied example of a DHI for Chinese Canadian PCa survivors, which is based on broader principles of collectivism and relationality from Indigenous and Black feminist theory. Our initial aim was to co-design a cultural adaptation of the *Ned* Clinic to provide compassionate care and meet the unmet needs of Chinese Canadian PCa survivors via digital health.

However, attending to cultural adaptation theory and the lived realities of settler colonialism identified gaps to interweave Indigenous and Black feminist teachings. We began by synthesizing design principles that surfaced as critical to our participants and their feelings of comfort and safety when receiving follow-up care. This allowed us to leverage digital health to strengthen relations between the survivor and their providers; improve accessibility to resources; and honor desires for relationality, accountability, and care [46,47]. Rather than adapting by defining Chinese Canadian culture, we co-designed to intervene in structural causes of health inequities created by settler colonial culture instead [21,48].

We applied *Etuaptmumk* by interweaving strengths from different ways of being and knowing, including those from Indigenous, Western, Chinese, and Black feminist traditions in relation to PCa follow-up and virtual care [27,30,31]. These included prioritizing relational care, accounting for the use of prostate-specific antigen screening as a recurrence monitoring tool, and the benefits of supportive care programs to create adaptation features [30,49]. The EPIC-CP validated questionnaire is a key part of clinical follow-up care, as it allows clinicians to identify possible areas of concern during follow-up [50]. The needs assessment addresses domains beyond clinical care, reflecting the holistic nature of the medicine wheel [51]. Access to resources includes education and guidance for the self-management of concerns across multiple domains. The app presents a “care contract” in the form of a schedule that clearly states the “terms” and dates of the user’s follow-ups [52]. It also respects the user’s privacy by providing access and allowing them to share their PHI on their terms [53]. Only key inputs are communicated for triage and response. Finally, language access is built into the app as a question of communication accessibility, rather than only culture.

This design approach and these features do not deny the fact that culture is a real influence and can be a source of strength in many peoples’ lives. However, we must go beyond implicating culture when designing DHIs for communities made vulnerable and instead address the overarching and underlying structures that create health inequities. Our design approach looked “up” at these structural causes rather than looking “down” and museumizing culture for participants through cultural sensitivity and competency. We demonstrate that a structural approach that applies teachings such as cultural safety and intersectionality can result in DHIs that are found to be acceptable, appropriate, and feasible for use while still leaving room for users to self-define and practice culture on their own terms. We are supporting, not replacing, the *labor* and *acts* of caring with digital health. Beginning with a paradigm shift opened a window to design for collective care, a scalable opportunity to benefit communities beyond Chinese Canadians with this *Ned Nurse* patient-facing app adaptation.

### Strengths and Limitations

We have created the first “cultural” adaptation of a PCa follow-up care application for Chinese Canadian survivors. We extended the accessibility of this prototype by offering it in 3 language versions and tested its validity through member checking by returning it to participants who had provided their experiences and expertise as part of the first phase of this project. The findings should be considered with some limitations. Our sample does not fully represent the Chinese Canadian PCa community, as the heterogeneity of the community makes it difficult to recruit a fully representative sample [42]. User testing did not differentiate between results derived from users who interacted with the app themselves and users who directed a facilitator to perform actions on their behalf. However, all participants received the same set of instructions to apply the think-aloud method. A broad description of our theoretical stance, setting and place, methods, and results are provided to enhance understanding. We think of and encourage the transferability of this research as to how it might be made meaningful (ie, valid) for other communities in places where they may be subject to similar constructs and patterns of oppression [32]. Finally, this study does not include the provider perspective, although *Ned* was developed with clinicians who provide follow-up care for patients from this community. Future studies should examine the clinician’s perspective on the design and development of similar DHIs, including provision of care through these apps, acceptability and feasibility, and implementation readiness.

### Conclusions

This study demonstrates the relationality of Indigenous and Black feminist ontologies, epistemologies, and methodologies to digital health design by providing a worked example of its empirical use for an adaptation of a PCa follow-up care app, the *Ned Nurse* Clinic, for Chinese Canadian PCa survivors. We applied UCD principles to develop a prototype design that supports the relational act of caring through digital technology by identifying structures that create inequities in the experiences of this community of survivors and designing to intervene and provide accessible, connected care instead. We hope that this prototype serves as a tool to help regenerate places of caring, as we have learned from Indigenous and Black feminist scholars’ teachings on power, place, and digital technologies.

## Appendix

Multimedia Appendix 1 Semistructured usability testing interview guide.

## Abbreviations

CBPR: community-based participatory research
COREQ: Consolidated Criteria for Reporting Qualitative Research
DHI: digital health innovation
EPIC-CP: Expanded Prostate Cancer Index Composite-Clinical Practice
GVRD: Greater Vancouver Regional District
PCa: prostate cancer
PHI: personal health information
UCD: user-centered design

## Data Availability

The data sets generated and analyzed during this study are available from the corresponding author upon reasonable request.
