# Supplementary material for: User-Centered Design and Usability of a Culturally Adapted Virtual Survivorship Care App for Chinese Canadian Prostate Cancer Survivors: Qualitative Descriptive Study
Source: JMIR Hum Factors. 2024 Jan 1;11:e49353. doi: 10.2196/49353 (PMC10790201; doi:10.2196/49353)
Supplement: Multimedia Appendix 1 [file humanfactors_v11i1e49353_app1.docx]

## Multimedia Appendix 1: Usability Testing Interview Guide

We are researchers from the University of Toronto. The objective of this study is to understand how virtual care can support Chinese Canadian prostate cancer survivors in the survivorship phase of their cancer trajectory. As part of this research, we are conducting two phases of interviews with people and caregivers of people living with prostate cancer. This usability test session is part of the second phase. Your time and insight are greatly appreciated.

We talked about your experiences with prostate cancer follow-up care and virtual care during Phase I of this study. We also spoke about whether you might feel comfortable or safe with having your follow-up care provided by a nurse over a virtual platform. Using this information, we created a prototype of a virtual follow-up care application for Chinese Canadian prostate cancer survivors like yourself. We call this application the *Ned* Clinic patient application.

We will show you how we have re-imagined the *Ned* patient application, and we will ask you to go through some hypothetical scenarios that you would encounter as a patient. You are welcome to ask any questions that may come to mind. First, do you have the MS Teams or Zoom application on your computer, or have you joined this meeting from your web browser?

*If yes* - The first thing I will do is share my screen which is linked to the current patient application prototype. Because you have MS Teams downloaded, I will be able to give you direct screen control so you can interact with the prototype directly.

*If no* – Due to the limitations with the browser version, I will be unable to give you screen control and will instead ask you to verbally state how you would approach each scenario. For example, you can tell me to scroll down or up, navigate to certain buttons, hover over anything, or click through to anything as you would be interacting with it. I will complete all of the actions for you instead.

As we go through the scenarios, please know that there are no wrong answers. Don’t worry about making mistakes! I’ll ask you to think aloud as much as possible, talk about what you’re looking at, and what you’re trying to do. This will be a big help to understanding how users interact with our application. Also, don’t worry that you’re going to hurt our feelings. We’re doing this to improve the application, so we need to hear your honest reactions.

Finally, please note that this is a prototype. That means it is not fully functional and there may be some bugs. We have pre-selected the answers to some questions for you and will let you know what these answers are. As we did previously, we would like to video-record this session. Are you comfortable if we do so? [wait for response] We will begin recording the session now.

**SCENARIO ONE: Asynchronous Visit Tasks**

*Precondition: User is on the patient app homepage with open tasks.*

*This is the page showing your follow-up care tasks. Every month, you will log-in to the Ned patient app to complete your tasks before a follow-up visit. There are two tasks. You must complete a wellness survey, called the EPIC-16. In addition to the wellness survey, you may also complete an optional needs assessment, which is meant to capture your holistic needs that may impact your quality of life. Once your tasks are completed, your clinician will be able to review your information through a dashboard and will manage your care as necessary.*

1. You have just landed on the Ned homepage to complete your tasks for your follow-up visit. Now please go ahead and begin the wellness survey.

- What are your thoughts on completing your tasks this way?
- How can *Ned* overall better support your holistic care needs (i.e. any considerations for the Ned technology? The service design)?

1. You have completed your tasks and have just received an automated clinician’s note. Please review your Clinician Note and Care Steps to complete your follow-up visit.

- What are your first impressions of the automated nurse’s note? Is the Clinician Note easy to read and understand? Is there anything you would change?

1. Finally, please go through and completing the needs assessment.

- What are your thoughts on the Ned Needs Assessment feature?
- Did you feel that these questions were easy to understand?
- Would you like to see any changes to these questions?

**SCENARIO TWO: My Care Profile**

*Precondition: User taps on My Care Profile in the navigation menu bar.*

1. You have landed on this page because you want to see more information regarding your follow-up care.
2. What do you think about the way that your follow-up care plan is presented (i.e. clear, concise, etc.)?
3. What may be missing that would be important for your follow-up care?
4. Please go ahead and review the “All Review Notes & My Notes” section.
5. Do you think this feature would be useful to you? Would you add your own notes to these Nurse’s Notes?

**SCENARIO THREE: Resources**

*Precondition: User taps on Resources in the navigation menu bar.*

*These are your educational resources, which are meant to give you an understanding of your disease. It includes information about your staging, treatment history, and follow-up care.*

1. You have landed on your resources page to look at information. Please explore this page and let us know when you would like to proceed.
2. What are your overall thoughts on this feature?
3. What do you like or not like about this feature?
4. Is there anything that might make it more likely for you to use these resources?
5. Care steps are instructions related to your care. For the purposes of our session, this is an example of the care steps you would receive. What are your thoughts regarding the Care Steps? Did you find the structure and included resources useful?

**SCENARIO FOUR: Chat**

*Precondition: User has completed the follow-up and initiated a message to the Clinician.*

*This is your communication page where you can chat with your clinician. The purpose of this chat is to help you address ad hoc issues that were not captured by either the wellness survey or needs assessment and may not require a synchronous follow-up. This scenario will ask you to explore this Ned chat feature in the app. For the purposes of testing, we have simulated the conversation and you can advance the screen by clicking directly on the prototype.*

1. You are a patient and have completed your wellness survey but would like to speak to the clinician about the clinic note you just received. Please walk us through how you would communicate this issue to the *Ned* Clinician.

- How did you find the overall process of communicating with the *Ned* automated support assistant?
- Was the distinction between the Automated Chatbot vs. Human Clinician Nathan clear? What could be improved?
- Did you enjoy using this *Ned* chat feature, or did you find it confusing to use? What would you like to see improved?

**SCENARIO FIVE: Labs & Imaging**

*Precondition: User taps on Labs & Imaging in the navigation menu bar.*

1. You would like to see a history of your Labs & Imaging. Please explore this page and let us know when you are comfortable to proceed.
2. What are your overall thoughts on this view?
3. Would you like to have your imaging results, such as your MRI or biopsy results, available to you? In a perfect world, what would that look like for you?
4. Is there anything you would add that would make this feature more useful to you?

**General Questions:**

1. What are your overall thoughts about the *Ned* app?

- Do you feel that you would be able to use this application? Would you prefer it to be in English or Chinese?
- Are there any additional features or tools you think would be helpful?
- Does anything about this app make you feel uncomfortable or anxious?

1. When do you think the best time to begin using this app might be (i.e. right after diagnosis, after finishing treatment, not at all)?
2. Would you be comfortable with your clinician providing your care through this app?

- If a nurse provided your care to you through this application, would you be comfortable with this?
- Would it be helpful if you had a navigator assigned (either trained peer or clinical navigator) to help you with your questions about the *Ned* Clinic?

*That’s it for my questions! We appreciate your time, willingness, and openness in talking with us today. Do you have any questions about our interview today and/or this study? In the next 2 weeks, we will be sending you an honorarium in the amount of $50.00 per hour in appreciation of your time. It is our hope that this study will be helpful to inform us on how best to provide and design care for patients like you in the future.*

*After we end our call, I will send the gift card to your email (confirm email address) along with a receipt form to confirm that you received the honorarium. Please fill out this receipt with your name and date and send it back to me. I will also be sending you a completed copy of the consent form, with my signature, for your records. If you have any questions after the study, please don’t hesitate to reach out by phone or email. Thank you for attending this session!*
